# Overexpression of NKG2D and IL24 in NK Cell-Derived Exosomes for Cancer Therapy

**DOI:** 10.3390/ijms26052098

**Published:** 2025-02-27

**Authors:** Chujun Huang, Qian Hu, Peiyun Wang, Mi Xie, Ying Zhang, Zhixing Li, Shuqing Tang, Yuxuan Zhang, Zhixin Tian, Xionghao Liu, Zhiqing Hu, Desheng Liang

**Affiliations:** 1 Center for Medical Genetics & Hunan Key Laboratory of Medical Genetics, School of Life Sciences, Central South University, Changsha 410078, China; huangchujun@sklmg.edu.cn (C.H.); huqian@sklmg.edu.cn (Q.H.); wangpeiyun@sklmg.edu.cn (P.W.); xiemi@sklmg.edu.cn (M.X.); zhangying@sklmg.edu.cn (Y.Z.); lizhixing@sklmg.edu.cn (Z.L.); tangshuqing@sklmg.edu.cn (S.T.); zhangyuxuan@sklmg.edu.cn (Y.Z.); tianzhixin@sklmg.edu.cn (Z.T.); liuxionghao@sklmg.edu.cn (X.L.); 2 MOE Key Lab of Rare Pediatric Diseases & Department of Cell Biology and Genetics, School of Basic Medical Sciences, Hengyang Medical School, University of South China, Hengyang 421001, China

**Keywords:** oncotherapy, exosome, IL24, NKG2D

## Abstract

Natural killer (NK) cell-derived exosomes (NK-Exos) are emerging as a promising avenue in cancer immunotherapy due to their inherent tumor-targeting properties and their capacity to deliver therapeutic agents directly to malignant cells. This research delves into the boosted anti-tumor potency of NK-Exos that has been genetically enhanced to overexpress NKG2D, a vital activating receptor, along with interleukin-24 (IL24), a cytokine renowned for its selective suppressive impact on tumor cells. NKG2D facilitates the recognition of tumor cells by binding to stress-induced ligands, while IL24 induces apoptosis and modulates immune responses to enhance tumor destruction. The NK-Exos engineered to express both NKG2D and IL24 significantly enhanced tumor targeting and increased the apoptosis rate of tumor cells by 30% in A549 and by 20% in HELA at 48 h compared with non-modified NK-Exos, respectively. Furthermore, this enhancement also impacted cell proliferation, with inhibition rates increasing by 30%, 15%, and 15% in A549, HELA, and MCF-7 cells, respectively, and it reduced A549 cell migration by 10%. The integration of NKG2D and IL24 within NK-Exos confers a dual therapeutic mechanism, synergistically amplifying their efficacy in cancer treatment. The utility of NK-Exos co-expressing NKG2D and IL24 offers a novel approach to overcome the limitations of current therapies, providing prolonged tumor suppression and precise targeting of malignant cells and holding great promise for clinical application.

## 1. Introduction

Exosomes are small extracellular vesicles (30–150 nm in diameter) released by a wide variety of cell types and are key players in intercellular communication [1]. They encapsulate a variety of bioactive molecules, including proteins, lipids, and nucleic acids, which are conveyed to recipient cells to modulate cellular functions [2]. Exosomes have garnered significant attention in cancer therapy due to their specificity for targeting cancer cells and delivering biologically active molecules directly to target cells [3]. Compared with other delivery systems, exosomes offer advantages including biocompatibility, low immunogenicity, and the capacity to traverse biological barriers, notably the blood–brain barrier, making them an ideal vehicle for therapeutic interventions [4].

In cancer therapy, exosomes are being explored both as biomarkers and as vehicles for delivering therapeutic agents, such as chemotherapeutic drugs, small interfering RNAs (siRNAs), and immune modulators [5]. Notably, exosomes derived from immune cells, especially natural killer (NK) cells, exhibit inherent anti-tumor properties, making them promising candidates for targeted cancer therapy. NK cells are a crucial component of the innate immune system, specializing in the recognition and elimination of malignant and virally infected cells without prior sensitization. NK cells exert their cytotoxic effects primarily through activating receptors, such as NKG2D, which recognizes stress-induced ligands on the surface of tumor cells, such as MICA, MICB, and ULBPs [6]. However, the expression of NKG2D on NK cells can be downregulated due to the influence of TGF-β, a cytokine secreted by tumor cells, which thereby curtails the efficacy of NK cells in targeting tumors [7,8]. Additionally, in certain solid tumors, NK cells are unable to directly contact the tumor cells due to the physical barriers imposed by the tumor stroma [9,10]. This can limit the NK cells’ access to their targets, thus reducing their cytotoxic potential within the tumor microenvironment [9]. To address this limitation, we can employ NK cell-derived exosomes (NK-Exos), which are non-cellular vesicles characterized with low immunogenicity and enhanced penetration capabilities owing to their small size. NK-Exos inherit the cytotoxic properties of their parent NK cells, and their ability to carry functional proteins, such as NKG2D, significantly enhances their tumor-targeting efficacy [11]. Equipped with NKG2D recognition sites, NK-Exos can directly target against tumor cells and are impervious to the immunosuppressive effects of TGF-β secreted from the tumor, allowing them to deliver NKG2D directly into the tumor microenvironment, where the interaction with NKG2D ligands initiates cytotoxic responses and induces tumor cell apoptosis [12,13]. Furthermore, NK-Exos possess the capability to deliver therapeutic agents effectively. The unique advantage of NK-Exos lies in their capacity to deliver anti-tumor agents to tumor cells with high specificity [13,14,15,16,17].

Enhancing the therapeutic potential of NK-Exos is pivotally achieved by engineering them to incorporate additional anti-tumor agents. Given the heterogeneity in most tumors, interleukin-24 (IL24), also known as melanoma differentiation-associated gene 7 (MDA-7), was chosen to be incorporated into the NK-Exos [18]. IL24 is a member of the IL10 cytokine family and has been shown to selectively induce apoptosis in tumor cells without affecting normal cells, and it is known for its bystander effects, inducing apoptosis in neighboring tumor cells as well [19]. IL24 exerts its anti-tumor effects through multiple mechanisms, including the induction of pro-apoptotic genes such as caspases, suppression of angiogenesis, and modulation of the tumor immune microenvironment [20].

Given the potent anti-tumor properties of both NKG2D and IL24, their combination with NK-Exos presents a novel and promising strategy for cancer therapy [11,14,21,22]. By engineering NK-Exos to overexpress NKG2D and IL24, these exosomes can simultaneously enhance the recognition of tumor cells via NKG2D–ligand interactions and induce apoptosis through IL24-mediated signaling pathways. The dual targeting of tumor cells via NKG2D engagement and IL24-induced apoptosis could potentially overcome the limitations of conventional therapies, such as drug resistance and off-target toxicity.

In this study, to assess the anti-tumor efficacy of NK-derived exosomes that overexpress both NKG2D and IL24, the NK cells expressing NKG2D and IL24 were constructed and NK-Exos were extracted. The tumor targeting, apoptosis induction, inhibition of tumor cell proliferation, and suppression of migration were examined. The anti-tumor efficacy of these engineered exosomes with NKG2D and IL24 was confirmed across a range of cancer cells, providing a novel approach for targeted cancer therapy.

## 2. Results

### 2.1. Identification of NKG2D-NK and IL24-NKG2D-NK Cell Lines

**Figure 1:**
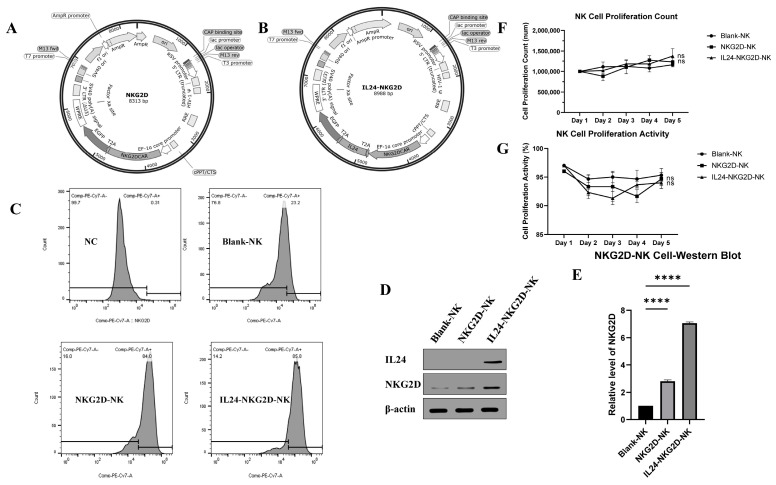
Lentiviral vector construction and identification of NKG2D-NK and IL24-NKG2D-NK cell lines. (**A**) NKG2D plasmid, (**B**) IL24-NKG2D plasmid, (**C**) flow cytometry detection of NKG2D overexpression, (**D**,**E**) Western blot (WB) analysis of NKG2D and IL24 expression, *n* = 5, (**F**) NK cell proliferation rate, and (**G**) NK cell viability, *n* = 3 (significance was determined using a one-way ANOVA followed by Tukey’s test; ns: not significant, **** *p* < 0.0001).

The NK92MI cells were transfected with lentivirus expressing NKG2D (Figure 1A), IL24-NKG2D (Figure 1B) to construct NKG2D-NK (NK92MI cell line with NKG2D-overexpressing), and IL24-NKG2D-NK (NK92MI cell line with NKG2D and IL24-overexpressing) cell lines. Flow cytometry was employed to assess the surface expression of NKG2D in the NK92MI cells (Figure 1C). The NKG2D-overexpressing groups, including NKG2D-NK and IL24-NKG2D-NK, showed significantly higher NKG2D expression compared with the Blank-NK control. This result confirmed that the lentiviral transduction was successful in these groups, and NKG2D expression levels were elevated as intended. Furthermore, the protein expression of NKG2D and IL24 in these cell lines was detected using a Western blot (WB) analysis (Figure 1D,E). Both the NKG2D-NK and IL24-NKG2D-NK groups exhibited significantly higher NKG2D expression compared with the Blank-NK group. The WB bands for NKG2D were markedly more intense in these groups, indicating robust expression. IL24 was detected only in IL24-NKG2D-NK cells. These results confirmed the successful overexpression of NKG2D and IL24.

The proliferation rates were comparable across all groups, including the Blank-NK control group (Figure 1F). The consistent proliferation rates across the experimental groups suggest that the overexpression of NKG2D and IL24 did not adversely affect NK92MI cell proliferation. These findings are crucial, as any significant deviations in proliferation could indicate cytotoxicity or transduction inefficiency.

NK92MI cell viability was evaluated using standard viability assays (Figure 1G). All groups maintained high viability, indicating that lentiviral transduction was generally well tolerated by NK92MI cells.

These results demonstrate the successful construction and characterization of NK92MI cell lines overexpressing NKG2D and both NKG2D and IL24. The strong expression of NKG2D in both the NKG2D-NK and IL24-NKG2D-NK groups indicates that these cells are well suited for further investigation into their potential therapeutic applications, particularly in terms of tumor recognition and immune response modulation.

### 2.2. Characterization of NK92MI-Derived Exosomes Post-Purification

**Figure 2:**
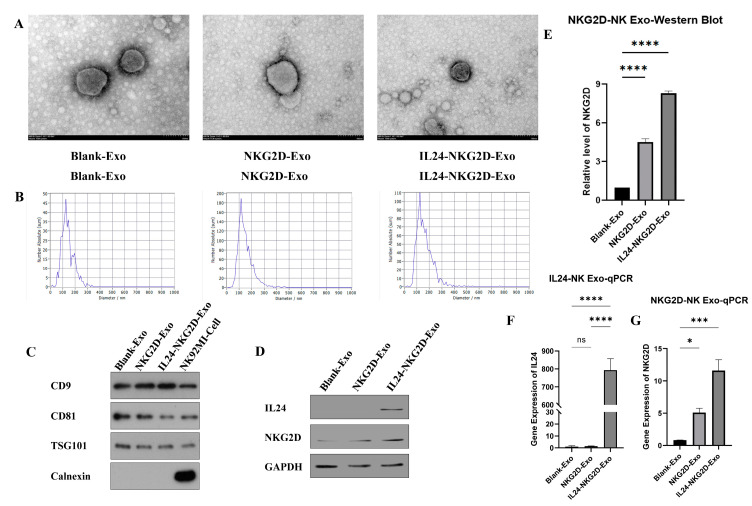
Characterization of NK-derived exosomes post-purification. (**A**) Transmission electron microscopy (TEM) for exosome morphology (scale bar = 100 nm), (**B**) nanoparticle tracking analysis (NTA) for exosome size distribution, (**C**) Western blot for exosome markers (CD9, CD81, TSG101, calnexin), (**D**,**E**) Western blot for IL24 and NKG2D in exosomes, *n* = 3, and (**F**,**G**) quantitative PCR (qPCR) for IL24 and NKG2D mRNA expression in exosomes, *n* = 3 (significance was determined using a one-way ANOVA followed by Tukey’s test; ns: not significant, * *p* < 0.05, *** *p* < 0.001, **** *p* < 0.0001).

Three groups of exosomes were obtained by isolating and purifying from three groups of NK92MI cell supernatants. TEM imaging confirmed the typical exosomal morphology in all three groups (Figure 2A). Each sample exhibited the characteristic cup-shaped vesicular structures with clear exosomal membranes. These results demonstrate that the structural integrity of the exosomes was preserved following isolation and purification, further supporting the successful exosome preparation. The NTA analysis demonstrated that the particle sizes of exosomes across all groups fell within the expected exosomal size range of 30–150 nm (Blank-Exo group: 121.57 ± 1.95 nm; NKG2D-Exo group: 120.33 ± 2.42 nm; IL24-NKG2D-Exo group: 129.33 ± 4.12 nm) (Figure 2B). All three groups displayed particle sizes consistent with the classical definition of exosomes, indicating that the exosome isolation procedure was successful, and the particle sizes were appropriate for further functional analysis. Nano-flow cytometry was employed to evaluate the purity of the isolated exosomes. The results showed that the exosome purity varied across the three groups (Blank-Exo group: 81.47%; NKG2D-Exo group: 83.91%; IL24-NKG2D-Exo group: 80.48%) (Table S1). The high purity levels confirm that the majority of particles that were isolated were indeed exosomes, with minimal contamination from other vesicles or debris.

A Western blot analysis was performed to assess the expression of classical exosome markers (CD9, CD81, TSG101) and the negative marker (calnexin) (Figure 2C). Calnexin, which is used to distinguish exosomes from cellular contamination, was only detected in the control group (NK92MI cell), indicating that the three groups (Blank-Exo, NKG2D-exo and IL24-NKG2D-exo) were devoid of cellular contamination, affirming the purity of the exosomal preparations. These findings confirm the successful isolation of pure exosomes in experimental groups, with minimal contamination by non-exosomal components.

Western blotting was also employed to detect the presence of IL24 and NKG2D within the exosomes (Figure 2D,E). IL24 expression was exclusively detected in the IL24-NKG2D-Exo group, consistent with the overexpression of IL24 in this group. Neither the Blank-Exo group nor the NKG2D-Exo group showed any detectable IL24 bands. NKG2D expression was detected in all three groups, though the expression was significantly higher in the experimental groups compared with the control group. The NKG2D band intensity was markedly higher in both the NKG2D-Exo group and the IL24-NKG2D-Exo group compared with the Blank-Exo group, indicating effective NKG2D overexpression. This result suggests that each group of exosomes inherits the NKG2D and IL24 characteristics of their NK92MI cells, respectively.

A qPCR analysis was conducted to quantify the mRNA expression levels of IL24 and NKG2D in exosomes (Figure 2F,G). IL24 mRNA expression was significantly elevated in the IL24-NKG2D-Exo group, with an approximate 750-fold increase compared with the Blank-Exo and NKG2D-Exo groups, both of which showed negligible IL24 mRNA expression. This result aligns with the overexpression of IL24 in the IL24-NKG2D-Exo group, confirming that IL24 was carried in the exosomes at both the protein and mRNA levels. NKG2D mRNA expression was significantly higher in both the NKG2D-Exo and IL24-NKG2D-Exo groups, with approximately 5-fold and 12-fold increases, respectively, compared with the Blank-Exo group. The higher expression in the IL24-NKG2D-Exo group further suggests that the co-expression of IL24 does not interfere with NKG2D expression and may even enhance its packaging into exosomes.

These results confirm the successful isolation, purification, and characterization of NK92MI cell-derived exosomes from control (Blank-Exo), NKG2D-overexpressing (NKG2D-Exo), and both NKG2D- and IL24-overexpressing (IL24-NKG2D-Exo) NK92MI cells, suggesting that the exosomes derived from the modified NK92MI cells are effectively carrying their intended molecular cargo, particularly in the IL24-NKG2D-Exo group, which showed enhanced levels of both IL24 and NKG2D.

### 2.3. Exosome Uptake, Localization, and Targeting Advantage of NKG2D Overexpression

A flow cytometric analysis was conducted to assess the expression of NKG2DLs, including hULBP-1, hULBP-2-5-6, hULBP-3, MICA, and MICB, in three tumor cell lines: A549 (lung adenocarcinoma), HELA (cervical cancer), and MCF-7 (breast cancer) (Figure S1). The varying expression levels of these ligands suggest differential recognition by NKG2D receptors on NK cells, influencing the targeting efficiency of NKG2D-overexpressing exosomes towards these tumor cells.

**Figure 3:**
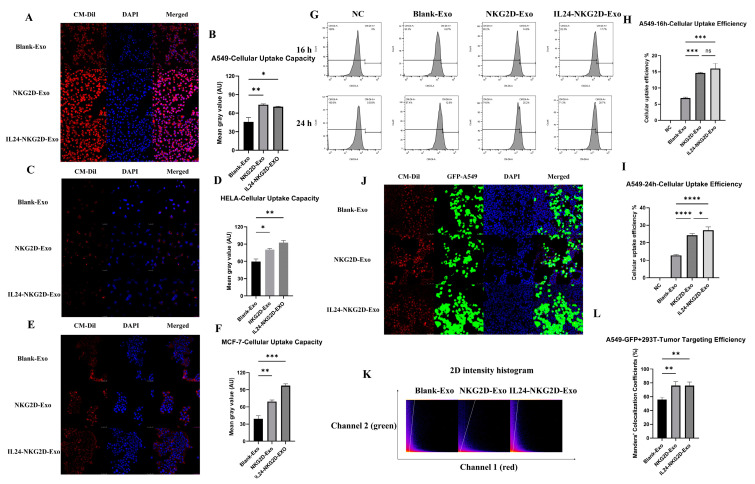
Exosome uptake, localization, and targeting advantage of NKG2D overexpression. (**A**–**F**) exosome uptake by tumor cells as assessed by fluorescence intensity (exosomes—red fluorescence (CM-Dil); nucleus—blue fluorescence(DAPI)): (**A**,**B**) A549, *n* = 3 (scale bar = 50 µm); (**C**,**D**) HELA, *n* = 3 (scale bar = 25 µm); (**E**,**F**) MCF-7, *n* = 3 (scale bar = 50 µm); (**G**–**I**) flow cytometric analysis of exosome uptake over time, *n* = 3; (**J**–**L**) GFP-A549 and 293T co-culture with exosomes (exosomes—red fluorescence (CM-Dil); GFP-A549—green fluorescence (GFP-A549); nucleus of GFP-A549 and 293T cells—blue fluorescence (DAPI)) (scale bar = 50 µm), Colocalization analysis: (**K**) Pearson’s correlation coefficient (PCC) scatterplots; (**L**) Manders’ colocalization coefficient, *n* = 3 (significance was determined using a one-way ANOVA followed by Tukey’s test; ns: not significant, * *p* < 0.05, ** *p* < 0.01, *** *p* < 0.001, **** *p* < 0.0001).

A co-culture experiment was performed in which A549, HELA, and MCF-7 cells were incubated with CM-Dil-labelled exosomes from three groups: Blank-Exo (control exosomes), NKG2D-Exo (NKG2D-overexpressing), and IL24-NKG2D-Exo (both NKG2D- and IL24- overexpressing) (Figure 3A–F). The uptake and intracellular localization of exosomes were analyzed by confocal microscopy, and the CM-Dil fluorescence intensity was quantified using ImageJ software. Both the NKG2D-Exo and IL24-NKG2D-Exo groups demonstrated significantly higher uptake by tumor cells compared with the Blank-Exo group. In A549 and HELA cells, the uptake of NKG2D-Exo and IL24-NKG2D-Exo exosomes was approximately 30% higher than the uptake of Blank-Exo. In MCF-7 cells, the uptake of NKG2D-Exo was approximately 30% higher, and IL24-NKG2D-Exo was approximately 60% higher than the Blank-Exo group. These results suggest that NKG2D-overexpressing exosomes have a clear targeting advantage, particularly in MCF-7 cells.

To assess the kinetics of exosome uptake, flow cytometry was performed to measure the uptake of CM-Dil-labelled Blank-Exo, NKG2D-Exo, and IL24-NKG2D-Exo by A549 cells at different time points (16 h and 24 h) (Figure 3G–I). At 16 h, A549 cells showed a 10% increase in the uptake of NKG2D-Exo and IL24-NKG2D-Exo compared with Blank-Exo (Figure 3H). At 24 h, the uptake of NKG2D-Exo and IL24-NKG2D-Exo was 18% higher than that of Blank-Exo (Figure 3I). These findings demonstrate that NKG2D-overexpressing exosomes that were uptaken by A549 significantly enhanced by time, confirming their superior tumor-targeting capability.

To further investigate exosome localization within tumor cells, GFP-A549 cells and non-tumor 293T cells were co-cultured with CM-Dil-labelled Blank-Exo, NKG2D-Exo, and IL24-NKG2D-Exo (Figure 3J–L). Confocal microscopy was used to capture the co-localization of exosomes (CM-Dil: red fluorescence) with tumor cells (GFP: green fluorescence), and colocalization was quantified using Fiji software (1.53c). A Pearson’s correlation coefficient (PCC) analysis showed positive correlation values (0 < PCC < 1) of red fluorescence and green fluorescence for all three groups, indicating that exosomes are indeed targeted to tumor cells. The colocalization scatterplots revealed strong alignment between red and green fluorescence signals, confirming the preferential targeting of exosomes to A549 cells (Figure 3K). Manders’ colocalization coefficient (M1) was used to calculate the proportion of red fluorescence (exosome signal) colocalized with green fluorescence (tumor cell signal) to the total red fluorescence. The colocalization coefficients for Blank-Exo, NKG2D-Exo, and IL24-NKG2D-Exo were approximately 56%, 76%, and 76%, respectively (Figure 3L). This suggests that NKG2D overexpression in NKG2D-Exo and IL24-NKG2D-Exo resulted in a 20% enhancement in tumor targeting compared with Blank-Exo. The positive colocalization between exosomes and A549 cells, particularly in the NKG2D-overexpressing groups, provides strong evidence of the tumor-targeting potential of NKG2D-engineered exosomes.

### 2.4. Cytotoxic Effect of NKG2D and IL24 Overexpressing Exosomes on Tumor Cells

**Figure 4:**
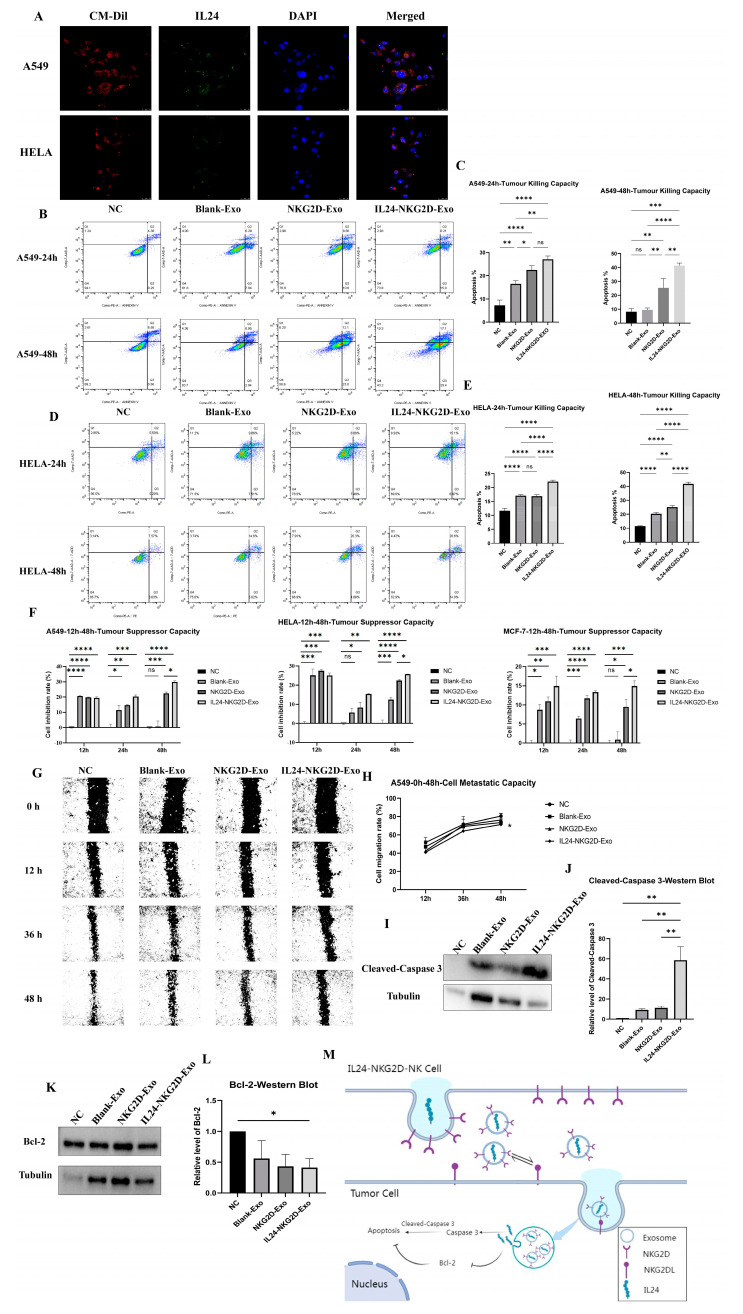
Cytotoxic effect of NKG2D and IL24 overexpressing exosomes on tumor cells. (**A**) Immunofluorescence verification of IL24 in IL24-NKG2D-Exo entering tumor cells (exosomes—red fluorescence (CM-Dil); IL24—green fluorescence (IL24), nucleus of A549 or HELA—blue fluorescence (DAPI)) (scale bar = 25 µm); (**B**–**E**) annexin V-FITC/PI flow cytometry for assessing apoptosis in tumor cells incubated with exosomes at 24 h and 48 h: (**B**,**C**) A549, *n* = 3; (**D**,**E**) HELA, *n* = 3; (**F**) CCK-8 assay for the growth inhibition of tumor cells incubated with exosomes at 12 h, 24 h, and 48 h: A549, HELA, MCF-7, *n* = 5. (**G**,**H**) Scratch assay to assess the impact of exosomes on tumor cell migration at 12 h, 36 h, and 48 h, *n* = 3 (scale bar = 200 µm); (**I**–**L**) Western blot analysis of apoptotic pathways: (**I**,**J**) cleaved caspase-3, *n* = 3; (**K**,**L**) Bcl-2, *n* = 3; (**M**) The mechanism schematic diagram (significance was determined using a one-way ANOVA followed by Tukey’s test; ns: not significant, * *p* < 0.05, ** *p* < 0.01, *** *p* < 0.001, **** *p* < 0.0001).

Immunofluorescence was used to visualize the internalization of IL24-loaded exosomes in A549 and HELA cells (Figure 4A). The IL24-NKG2D-Exo were labelled with CM-Dil (red fluorescence), while IL24 was recognized by the corresponding antibody (displayed as green fluorescent), and DAPI was used to stain the nucleus (blue fluorescence). The confocal images showed that tumor cells took up the IL24-NKG2D-Exo, as indicated by the red fluorescence of CM-Dil. Furthermore, green fluorescence corresponding to IL24 was detected in the cytoplasm of the A549 and HELA cells. The co-localization of red and green fluorescence within the tumor cells indicates that the IL24-NKG2D-Exo successfully delivered IL24 protein into A549 and HELA cells. This confirms the potential of these exosomes to act as delivery vehicles for therapeutic proteins, facilitating intracellular functions that could contribute to tumor suppression.

To assess the cytotoxic effects of exosomes, A549 and HELA cells were co-cultured with Blank-Exo, NKG2D-Exo, and IL24-NKG2D-Exo for 24 h and 48 h, respectively. Annexin V-FITC/PI flow cytometry was performed to evaluate apoptosis in these cells (Figure 4B,D). At 24 h, the apoptosis rates of A549 cells in the Blank-Exo, NKG2D-Exo, and IL24-NKG2D-Exo groups were significantly higher than the NC group (negative control, no exosome treatment) (Figure 4C). The apoptosis rates in the NKG2D-Exo and IL24-NKG2D-Exo groups were also significantly higher than that in the Blank-Exo group, although there was no significant difference between NKG2D-Exo and IL24-NKG2D-Exo. At 48 h, no significant difference in apoptosis was observed between the Blank-Exo and NC groups, while the apoptosis rates of NKG2D-Exo and IL24-NKG2D-Exo were significantly higher (Figure 4C). The apoptosis rate in NKG2D-Exo was approximately 15% higher than that in the NC group and Blank-Exo group, while IL24-NKG2D-Exo exhibited a 30% higher rate. Furthermore, the IL24-NKG2D-Exo group showed a 15% higher apoptosis rate compared with NKG2D-Exo. After 24 h, the apoptosis rates of HELA cells in Blank-Exo, NKG2D-Exo, and IL24-NKG2D-Exo exhibited significantly higher apoptosis rates than that in the NC group (Figure 4E). The IL24-NKG2D-Exo group demonstrated a significantly higher apoptosis rate compared with both the Blank-Exo and NKG2D-Exo groups. At 48 h, all three exosome groups (Blank-Exo, NKG2D-Exo, IL24-NKG2D-Exo) displayed significantly higher apoptosis rates than the NC group (Figure 4E). The apoptosis rate in NKG2D-Exo was approximately 15% higher than in the NC group and 5% higher than in the Blank-Exo group. The IL24-NKG2D-Exo group exhibited a 30% higher apoptosis rate than the NC group and a 20% higher rate than the Blank-Exo group. Importantly, IL24-NKG2D-Exo induced 15% more apoptosis than NKG2D-Exo. These results suggest that exosomes overexpressing NKG2D, and especially those also overexpressing IL24, enhance the induction of apoptosis in tumor cells over time. The significant increase in apoptosis rates in the IL24-NKG2D-Exo group compared with the NKG2D-Exo and Blank-Exo groups indicates that IL24 plays a crucial role in promoting apoptosis. While NKG2D overexpression enhances cytotoxicity compared with the Blank-Exo group, the combination with IL24 further amplifies this effect compared with the NKG2D-Exo group, potentially through synergistic mechanisms that activate apoptotic pathways more effectively.

The CCK-8 assay was used to evaluate the growth inhibition of A549, HELA, and MCF-7 cells following co-culture with exosomes for 12 h, 24 h, and 48 h (Figure 4F). In the CCK-8 assay of the A549 cell, all exosome-treated groups showed significantly higher inhibition of A549 cells growth compared with the NC group at 12 h and 24 h. There was no significant difference in inhibition observed between the Blank-Exo and NC groups, while the NKG2D-Exo and IL24-NKG2D-Exo groups displayed significantly higher inhibition rates (20% and 30%, respectively) compared with the NC group and Blank-Exo group at 48 h. Regarding HELA cells, all exosome-treated groups exhibited significant growth inhibition compared with the NC group at 12 h. At 24 h, the NKG2D-Exo and IL24-NKG2D-Exo groups showed significantly higher inhibition than the NC group, while no significant difference was observed between the Blank-Exo and the NC group. At 48 h, the NKG2D-Exo and IL24-NKG2D-Exo groups exhibited significantly higher inhibition than the NC group (20% and 25%, respectively). And in MCF-7 cells, at 12 h and 24 h, all exosome-treated groups showed significantly higher inhibition compared with the NC group. At 48 h, the NKG2D-Exo and IL24-NKG2D-Exo groups demonstrated significant inhibition of MCF-7 cells (9% and 15%, respectively), while no significant difference was observed between the Blank-Exo and NC groups. Additionally, the IL24-NKG2D-Exo group showed a better inhibitory effect on tumor growth than the NKG2D-Exo group at 48 h among the three types of cells. The CCK-8 assay results indicate that IL24-NKG2D-Exo can effectively inhibit tumor cell growth over extended periods. The enhanced inhibitory effects observed in the IL24-NKG2D-Exo group suggest that the presence of IL24 augments the anti-proliferative activity of the exosomes. Notably, the combination of NKG2D and IL24 had a more pronounced effect on A549 and HELA cells over time, highlighting the potential of this strategy in targeting different tumor types.

A scratch wound healing assay was used to evaluate the impact of NK-Exo on A549 cell migration. The wounds were created at 0 h, and the scratch closure was monitored at 12 h, 36 h, and 48 h after co-culturing with Blank-Exo, NKG2D-Exo, and IL24-NKG2D-Exo exosomes (Figure 4G). At 48 h, the NC group exhibited an approximately 80% cell migration rate, whereas there was a significant inhibition of cell migration in the IL24-NKG2D-Exo group, with a 10% decrease in the cell migration rate (Figure 4H). The wound healing assay demonstrates that IL24-NKG2D-Exo significantly inhibits the migratory ability of A549 cells. The lack of wound closure in the IL24-NKG2D-Exo group suggests that IL24 may contribute to anti-migratory effects, potentially by modulating signaling pathways involved in cell motility. The partial inhibition observed in the NKG2D-Exo group indicates that NKG2D overexpression alone has some impact on migration, but the effect is substantially enhanced when combined with IL24.

To further investigate the apoptotic mechanisms, a Western blot analysis was performed to detect cleaved caspase-3 and Bcl-2 expressions in A549 cells co-cultured with Blank-Exo, NKG2D-Exo, and IL24-NKG2D-Exo (Figure 4I–L). The expression of cleaved caspase-3 was significantly upregulated in the IL24-NKG2D-Exo group, approximately 60-fold higher than that in the NC group and 50-fold higher than that in the Blank-Exo and NKG2D-Exo groups. All exosome-treated groups displayed significant downregulation of the anti-apoptotic protein Bcl-2 compared with the NC group, indicating apoptosis induction.

These results confirm that IL24-NKG2D-Exo effectively induces apoptosis via caspase-3 activation, supporting its enhanced tumoricidal capacity compared with NKG2D-Exo and control exosomes. The substantial increase in cleaved caspase-3 expression in the IL24-NKG2D-Exo group indicates robust activation of the apoptotic pathway. Cleaved caspase-3 is a critical executor of apoptosis, and its elevated levels suggest that the exosomes effectively induce programmed cell death in tumor cells. The concurrent downregulation of Bcl-2, an anti-apoptotic protein, further supports the pro-apoptotic effect of the exosomes. The data imply that the overexpression of IL24 enhances the activation of apoptotic mechanisms, potentially through intrinsic pathways, leading to increased tumor cell susceptibility to exosome-mediated cytotoxicity.

The collective findings from these experiments demonstrate that IL24-NKG2D-Exo possess enhanced cytotoxic effects against tumor cells compared with NKG2D-Exo or control exosomes. A mechanism schematic diagram was drawn to illustrate the exosomal NKG2D receptor, which interacted with NKG2D ligands on tumor cells, and the IL24-mediated apoptotic pathways (Figure 4M). The overexpression of NKG2D improves the targeting and binding of exosomes to tumor cells expressing NKG2D ligands, facilitating increased uptake. The addition of IL24 appears to amplify the anti-tumor effects by promoting apoptosis, inhibiting cell proliferation, and reducing migratory capacity. These results highlight the potential of using genetically modified NK cell-derived exosomes as a therapeutic strategy for cancer treatment. The dual overexpression of NKG2D and IL24 enhances the specificity and efficacy of exosome-mediated anti-tumor activity, offering a promising avenue for the development of novel oncotherapy approaches.

## 3. Discussion

The findings from this study provide significant insights into the anti-tumor effects of NK-Exo, particularly those overexpressing NKG2D and IL24. These exosomes demonstrated enhanced tumor cell killing through multiple mechanisms, including increased apoptosis, growth inhibition, and suppression of tumor cell migration. The results also indicate potential interactions between IL24 and NKG2D signaling pathways, which may further augment the therapeutic potential of these engineered exosomes.

NK cells are a cornerstone of the innate immune system, recognized for their ability to identify and destroy tumor cells through the activation of receptors such as NKG2D [23]. While NK cell-based therapies have shown promise in preclinical and clinical settings, a major challenge for NK cell therapy is the physical and immunosuppressive barriers posed by the tumor microenvironment (TME) [24]. In solid tumors, the stromal components and chemokines (e.g., CCL27, CXCL12) often prevent the infiltration of NK cells into the tumor core, limiting their ability to exert cytotoxic effects [9,25,26]. In contrast, exosomes, due to their small size (30–150 nm), can penetrate deep into the TME and deliver therapeutic payloads directly to tumor cells [11,17,27]. This enhanced penetrative ability makes NK-Exos a superior choice for targeting solid tumors. Moreover, NK-Exos have been experimentally verified to inherit NKG2D receptors from NK cells and were expressed on exosomal membranes [11,14,21]. Direct NK cell therapies are primarily reliant on the endogenous cytotoxic mechanisms of NK cells, which may not be sufficient against highly aggressive tumors [9,28]. By engineering NK-Exos to carry IL24 and overexpressing NKG2D, the therapeutic potential is significantly enhanced. IL24 induces selective apoptosis in tumor cells through multiple pathways, including the activation of pro-apoptotic genes and modulation of immune responses [20]. Meanwhile, NKG2D on exosomes facilitates tumor cell targeting by recognizing stress-induced ligands such as MICA, MICB, and ULBP, which are commonly overexpressed in tumors [12,29]. The combination of these molecules within exosomes provides a dual mechanism of action, enhancing both targeting specificity and cytotoxic efficacy. Furthermore, exosomes are less susceptible to immune evasion strategies employed by tumors, such as the secretion of immunosuppressive factors (e.g., TGF-β) that inhibit NK cell activity [30]. One concern with NK cell-based therapies is that long-term exposure to NKG2D ligands derived from tumor exosomes in TME leads to impaired cytotoxicity and reduced tumor immune surveillance [31,32,33]. NK-Exo, when engineered with both IL24 and NKG2D, exhibit enhanced tumor selectivity and killability due to the tumor-specific properties of IL24 and the ability of NKG2D to target highly overexpressed ligands on tumor cells, making NK-Exos a more effective and safer therapeutic alternative.

One notable observation from this study is the potential role of IL24 in enhancing NKG2D expression within exosomes. The qPCR and Western blot results demonstrated that IL24-NKG2D-Exo exhibited significantly higher NKG2D expression compared with NKG2D-Exo. Although there have been no direct reports of interactions between IL24 and NKG2D, it is speculated that IL24 may indirectly contribute to the upregulation of NKG2D, potentially via the IL24/STAT3 signaling pathway. Previous research has indicated that IL24 can activate STAT3, leading to its phosphorylation (pSTAT3), which in turn may upregulate NKG2D expression through downstream transcriptional effects [34,35].

The STAT3 pathway is known to play a key role in immune regulation and tumor suppression. In the context of NKG2D-expressing exosomes, the enhanced expression of NKG2D driven by IL24 could improve the ability of NK-Exos to recognize and bind to tumor cells expressing NKG2D ligands (NKG2DLs) (Figure 3H,I). As tumor cells such as A549, HELA, and MCF-7 frequently overexpress these ligands, the augmented targeting ability of IL24/NKG2D-co-expressing exosomes could contribute to more effective tumor cell killing.

Interestingly, the apoptosis data revealed no significant difference between the NKG2D-Exo and IL24-NKG2D-Exo groups at 24 h in A549 cells, suggesting that NKG2D plays a dominant role in early tumor cell targeting and apoptosis induction. However, by 48 h, the IL24-NKG2D-Exo group exhibited a significantly higher apoptotic rate compared with NKG2D-Exo, indicating that IL24 plays a more prominent role in sustaining apoptosis over time. This delayed effect of IL24 may be due to the activation of downstream signaling pathways, which can take longer to manifest [22].

The wound healing assay provided further evidence of the anti-tumor capabilities of IL24-NKG2D-Exo. After 48 h, A549 cells in the IL24-NKG2D-Exo group exhibited low migration into the wound area, compared with partial wound closure in the Blank-Exo and NKG2D-Exo groups. This suggests that the combination of NKG2D and IL24 is particularly effective at inhibiting the migratory potential of tumor cells. IL24 has been shown to suppress tumor invasion and metastasis by modulating matrix metalloproteinase (MMP) expression [36] or SDF-1/CXCR4 signaling axis [37], while NKG2D enhances the targeting and killing of tumor cells, further reducing their ability to migrate.

In the current study, the effects of IL24-NKG2D-Exo on cell viability were investigated using the CCK-8 assay, focusing particularly on the differential responses between non-tumor cells (293T) (Figure S2) and tumor cells (A549, HELA, MCF-7) (Figure 4F). The results demonstrated that, in contrast to the tumor cells, 293T cells exhibited an inverse trend in growth inhibition over 24 h and 48 h following exposure to IL24-NKG2D-Exo, and there was no significant difference in cell inhibition rate compared with NC groups at 48 h. This trend suggests that IL24-NKG2D-Exo selectively targets tumor cells for growth suppression, while exerting minimal or no harmful effects on non-tumor cells. The differential response between non-tumor and tumor cells is critical in evaluating the safety and potential therapeutic application of IL24-NKG2D-Exo in cancer treatment [38].

These findings are consistent with previous studies that have demonstrated the safety of IL24-based therapies. For instance, it has been reported that IL24 induces apoptosis selectively in tumor cells, sparing normal cells by acting through specific pathways that are regulated in malignancies [18,39]. Similarly, NK-Exos have been shown to exhibit tumor-specific cytotoxicity, with minimal effects on healthy cells, suggesting a favorable safety profile for clinical applications.

While our study provides strong in vitro evidence demonstrating the enhanced anti-tumor efficacy of IL24-NKG2D-Exo, the absence of in vivo validation remains a key limitation. Tumor progression and immune interactions in vivo are significantly influenced by factors such as the tumor microenvironment, immune cell infiltration, and systemic exosome biodistribution, which cannot be fully replicated in vitro [15,16]. Therefore, while our findings indicate that IL24-NKG2D-Exo can effectively target and induce apoptosis in tumor cells, it remains essential to evaluate their therapeutic potential in an in vivo system to confirm their efficacy, safety, and long-term stability. Additionally, evaluating the immune response and potential off-target effects in vivo will be crucial for further clinical translation. Future studies will focus on in vivo experiments to assess the biodistribution, pharmacokinetics, and anti-tumor effects of these engineered exosomes. By incorporating these in vivo investigations, we aim to bridge the gap between our current in vitro findings and the eventual development of a robust exosome-based therapeutic strategy for cancer treatment.

## 4. Materials and Methods

### 4.1. Generation and Characterization of Stable NKG2D-NK and IL24-NKG2D-NK Cells

The human NKG2D gene (GenBank: NM_007360) and IL24 gene (GenBank: NM_006850) were amplified by PCR using high-fidelity DNA polymerase (Thermo Fisher Scientific, Waltham, MA, USA). Restriction sites suitable for insertion into the multiple cloning site (MCS) of the lentiviral transfer vector (pRRLSIN.cPPT.PGK-GFP.WPRE, addgene 12252, Cambridge, MA, USA) were incorporated into the primers. The amplicons were digested with Age I and BamH I, purified, and ligated into the pLenti transfer vector. Ligation products were transformed into Stbl3 competent *E. coli* cells. Plasmids from positive colonies were purified using E.Z.N.A^®^ Plasmid Mini Kit (Omega D6945-01, Doraville, GA, USA). HEK293T cells were seeded in 10 cm dishes at a density of 3 × 10⁶ cells per dish and incubated overnight. The cells were transfected with a mixture of 10 µg of transfer plasmid (pLenti-NKG2D or pLenti-NKG2D-IL24), 7.5 µg of psPAX2, and 4 µg of pMD2.G using Lipofectamine™ 2000 (Invitrogen™, Carlsbad, CA, USA) according to the manufacturer’s protocol. The supernatant containing lentiviral particles were collected at 30 and 54 h post-transfection, filtered through a 0.45 µm filter, and concentrated by ultracentrifugation at 80,000× *g* for 3 h at 4 °C. The viral pellet was resuspended in 1 mL of PBS and subpackage stored at −80°C. NK92MI cells (ATCC CRL-2408 ™, Manassas, VA, USA) were cultured to a density of 5 × 10^5^ cells/mL and resuspended in complete Alpha MEM (Gibco, Grand Island, NY, USA). Cells were then transferred to a 10 cm dish (1 × 10^6^ cells/mL) in 10 mL of fresh medium supplemented with 8 µg/mL polybrene to enhance transduction efficiency. Concentrated lentiviral particles were added to the NK92MI cells at a multiplicity of infection (MOI) of 100. Cells were incubated at 37 °C in 5% CO_2_. After transduction, the expression of EGFP was observed by a microscope, and cells were taken every 3 days for cell counting using trypan blue staining. Based on the cell counts, fresh culture medium was added to adjust the cell density to between 5 × 10^5^ cells/mL and 1 × 10^6^ cells/mL. When stabilization of EGFP expression was observed, NKG2D expression was detected by flow cytometry (Cytek^®^ Aurora, Fremont, CA, USA) using PE/Cyanine 7 anti-human CD314 (NKG2D) antibody (BioLegend 320812, San Diego, CA, USA) to determine transduction efficiency and expression levels.

### 4.2. Generation of Stable GFP-A549 Cells

A549 (lung adenocarcinoma, ATCC CCL-185™, Manassas, VA, USA) cells were seed in 6-well plates at a density of 1 × 10^5^ cells/well and incubate overnight; when the cells were 50–60% confluent, the lentiviral virus (SV40T GFP-puromycin, HANBIO, Shanghai, China) was added to each well of A549 cells. We incubated the cells at 37 °C in 5% CO_2_ for 24 h, then replaced the medium with fresh Dulbecco’s Modified Eagle Medium (DMEM) (Gibco, Grand Island, NY, USA). After 48 h, the transduced cells were selected with puromycin at a final concentration of 2 µg/mL. We replaced the medium every 2 days, continuing the puromycin selection until only GFP-positive A549 cells remained.

### 4.3. Cell Culture

293T (kidney epithelial-like cell, ATCC CRL-3216™, Manassas, VA, USA), A549, GFP-A549, HELA (cervix Adenocarcinoma, ATCC CCL-2™, Manassas, VA, USA), and MCF-7 (breast Adenocarcinoma, ATCC HTB-22™, Manassas, VA, USA) were cultured in DMEM supplemented with 10% FBS (Gibco, Grand Island, NY, USA). Cells were passaged at the 80% confluence by washing with Dulbecco’s phosphate-buffered saline (DPBS) (Gibco, Grand Island, NY, USA), followed by detachment using 0.05% trypsin-EDTA (Gibco, Grand Island, NY, USA) for 3 min at 37 °C. The trypsin was neutralized with culture medium, and the cells were centrifuged at 1000 rpm for 5 min before resuspending in fresh medium. Blank-NK (non-modified), NKG2D-NK (overexpressing NKG2D), IL24-NKG2D-NK (overexpressing both IL24 and NKG2D) cells were cultured in serum-free NK92 cell medium (TBD, Tianjin, China), maintained at 37 °C in a humidified incubator with 5% CO_2_. The medium was replaced every 3 days, and the cells were centrifuged at 700× *g* for 5 min to isolate cells and the culture medium, which was used to isolate exosomes.

### 4.4. Extraction and Characterization of NK-Derived Exosomes

After 72 h of Blank-NK, NKG2D-NK, and IL24-NKG2D-NK cells culturing, the conditioned mediums were collected by centrifuging the culture at 700× *g* for 5 min to remove the cells. The conditioned mediums were subjected to a series of centrifugation steps to remove cell debris, apoptotic bodies, and larger vesicles: 2000× *g* for 20 min to eliminate cell debris; and 10,000× *g* for 30 min to pellet larger vesicles and apoptotic bodies. Filter the supernatant through a 0.22 µm filter to remove particles larger than exosomes. The filtered supernatants were transferred to ultrafiltration tubes (Merck Millipore Amicon^®^ Ultra-15 Centrifugal Filters Ultracel^®^-100K, Billerica, MA, USA) and ultrafiltrated at 3000× *g* for 20 min at 4 °C and washed with DPBS three times. Following this step, the supernatants of collection tube were discarded, and the exosome of the concentration tube were collected, to which we added exosome isolation reagent (Invitrogen 4478359, Carlsbad, CA, USA) overnight at 4 °C. After centrifugation, the supernatant was discarded, and the exosome pellet was resuspended in DPBS for downstream analysis or storage at −80 °C.

For morphological characterization, a small aliquot of purified exosomes was adsorbed onto a copper grid and negatively stained with uranyl acetate. The grid was air-dried and examined using transmission electron microscopy (TEM) to visualize the typical cup-shaped morphology of exosomes.

The size distribution and concentration of exosomes were determined using a nanoparticle tracking analysis (NTA). The exosome suspension was diluted in PBS to the appropriate concentration and analyzed using an NTA system, which provides data on the particle size and number.

Exosome identification was confirmed by a Western blot analysis of characteristic exosomal markers. Protein samples were subjected to SDS-PAGE and transferred to a PVDF membrane. The membrane was probed with antibodies against known exosome positive markers: CD9, CD81, and TSG101; and a negative marker: calnexin. The purity of the exosomes was assessed using a nanoparticle flow cytometer. By measuring the particle count of exosomes and the solvent (DPBS) before and after the addition of Triton, the purity of the exosomes was calculated as shown in Table S1.

For labelling CM-Dil, the purified exosomes were resuspended in 200 µL of PBS. An amount of 1 µL of the 2 µM CM-Dil (Life technologies CellTracker^TM^ C7000, Invitrogen™, Carlsbad, CA, USA) was added to the exosome suspension, ensuring that the dye was in excess relative to the exosome lipid content. The solution was gently mixed by pipetting up and down to ensure even distribution of the dye. The exosome–dye mixture was incubated at 37 °C for 20 min in the dark. Exosome isolation reagent (Invitrogen 4478359, Carlsbad, CA, USA) was added overnight at 4 °C and centrifugation. After centrifugation, the supernatant was discarded, and the Dil–exosome pellet was resuspended in DPBS for NTA.

### 4.5. Western Blot

The cell pellets were washed twice with cold DPBS and resuspended in ice-cold RIPA buffer (Beyotime^®^ P0013B, Shanghai, China) containing a protease and phosphatase inhibitor cocktail. The resuspended cells were incubated on ice for 30 min, then sonicated briefly (3 bursts of 5 s each). Protein and extracted exosome concentration were determined using a BCA protein assay kit (Thermo Scientific™ Pierce 23225, Waltham, MA, USA) according to the manufacturer’s instructions. Equal amounts of protein or exosomes were mixed with 4× loading buffer and heated at 95 °C for 5 min to denature the proteins. The samples were loaded onto a 10% SDS-PAGE gel (Beyotime^®^ SDS-PAGE Gel Quick Preparation Kit P0012AC, Shanghai, China). Following electrophoresis, proteins were transferred onto a PVDF membrane using a wet transfer system. The membranes were blocked in 5% bovine serum albumin (BSA) in Tris-buffered saline with 0.1% Tween-20 (TBST) for 1 h at room temperature, then incubated with the appropriate primary antibodies diluted in TBST containing 5% BSA: Anti-NKG2D (Abcam ab96606, Cambridge, MA, USA), Anti-IL24 (Clone Clound PAC064Hu01, Wuhan, China), Anti-β-actin (Zen-bioscience R23613, Chengdu, China), Anti-CD9 (Abcam ab275018, Cambridge, MA, USA), Anti-CD81 (Abcam ab275018, Cambridge, MA, USA), Anti-TSG101(Abcam ab275018, Cambridge, MA, USA), Anti- calnexin (Abcam ab275018, Cambridge, MA, USA), Anti-GAPDH (Sigma-Aldrich G9295, St. Louis, MO, USA), Anti-cleaved caspase-3 (Cellsignaling #9661, Danvers, MA, USA), Anti-Bcl-2 (Affinity Biosciences AF6139, Cincinnati, OH, USA‌), and Anti-tubulin (Sigma-Aldrich T6199, St. Louis, MO, USA) overnight at 4 °C on a shaker. The membranes were washed 3 times, then incubated with HRP-conjugated secondary antibodies for 1 h at room temperature and washed 3 times with TBST. The membranes were incubated with ECL substrate (Zen-bioscience 17046, Chengdu, China). The protein bands were visualized using a chemiluminescence detection system (Bio-Rad, Hercules, CA, USA). The bands were quantified using image analysis software (Image J 1.53e).

### 4.6. RNA Extraction and Quantification in Exosomes

Total RNA from the exosomes was extracted by an RNA extraction kit (Transgen *EasyPure^®^* ER601-01-V2, Beijing, China), and a qRT-PCR reagent kit (Transgen *PerfectStart^®^* AUQ-01, Beijing, China) was used for a real-time quantitative PCR with the CFX96 Real-Time System (Bio-Rad, Hercules, CA, USA). The threshold cycle (Ct) values were calculated for each sample and normalized via GAPDH. The relative expression of the target RNA could be calculated using the 2^−ΔΔCt^ method. The sequences of primers are listed in Table S2.

### 4.7. Exosome Uptake

A549 cells were seeded into 12-well plates at a density of 5 × 10^4^ cells per well in DMEM supplemented with 10% FBS and incubated for 24 h. The medium was aspirated from A549 cells and washed with DPBS. Dil–Exosomes were added to each well and incubated at 37 °C for various time intervals (16 h, and 24 h) to assess time-dependent exosome uptake by flow cytometry.

### 4.8. Co-Culture with Exosomes

Cells (A549, HELA, MCF-7, GFP-A549 and 293T) were seeded onto sterile glass coverslips placed in separate wells of a 12-well plate at a density of 2 × 10^5^ cells per well. The cells were cultured in DMEM supplemented with 10% FBS for 24 h. The medium was aspirated from cells and washed once with DPBS. The same amount of Dil–exosomes suspension was added to each well, then incubated for 14 h at 37 °C. The cells were washed three times with DPBS, then fixed by adding 500 µL of 4% paraformaldehyde (PFA) to each well and incubated for 15 min at room temperature. The cells were washed three times with DPBS to remove excess PFA. An amount of 300 µL of DAPI solution (Boster AR1176, Wuhan, China) was added to each well for 5 min to stain the cell nuclei. The cells were washed three times with DPBS to remove excess DAPI. Used a confocal microscope with a 63× oil-immersion objective to visualize the cells. Set microscope filters for detecting CM-Dil, GFP, and DAPI.

### 4.9. Quantitative Analysis of Average Fluorescence Intensity

In Image J (1.53e), after opening the image, a single channel was extracted (Image-Color-Split Channels). The threshold was adjusted, and the appropriate area was selected (Image-Adjust-Threshold). An appropriate threshold algorithm was selected (Image-Adjust-Auto Threshold). The parameters were set to be measured (Analyze-Set Measurements) and detected (Analyze-Measure). “Mean” in results means the mean gray value, which is the ratio of integrated density to area.

### 4.10. Colocalization Analysis [40,41,42]

In Image J (1.53c), after opening the image, the channel was separated, leaving the two channels with the target protein (Image-Color-Split Channels). False colors were added to each channel (Image-Color-Channels Tool). The Colorc 2 plugin was opened for computation (Analyze-Colocalization-Coloc 2). Both channels were selected, and both algorithms were checked, for which we clicked OK. In result, a 2D intensity histogram was selected to obtain the scatterplot.

Pearson’s correlation coefficient (PCC): The value of PCC ranges from 1 to −1. A value of 1 means perfect correlation; −1 indicates completely negative correlation, and 0 indicates a random relationship (fluorescence A and fluorescence B are randomly distributed, with no correlation).

$$\mathrm{PCC}=\frac{\sum_{i}\left(R_{i}-\overset{-}{R})\times (G_{i}-\overset{-}{G}\right)}{\sqrt{\sum_{i}{\left(R_{i}-\overset{-}{R}\right)}^{2}\times \sum_{i}{\left(G_{i}-\overset{-}{G}\right)}^{2}}}$$

Manders’ colocalization coefficients (MCCs): M1 and M2 represent the portion of one fluorescence co-located with another fluorescence, accounting for the proportion of the total amount of this fluorescence. It is equivalent to determining the overlap ratio of two fluorescent molecules. For example, M1 in the formula represents the proportion of the red fluorescent part co-located with green fluorescence to the total red fluorescent region.

$$M_{1}=\frac{\sum_{i}R_{i,\mathrm{colocal}}}{\sum_{i}R_{i}}$$

### 4.11. Immunofluorescence Verification of IL24 in IL24-NKG2D-Exo Entering Tumor Cells

A549 and HELA cells were seeded on sterile glass coverslips placed in 12-well plates at a density of 5 × 10^4^ cells per well. Dil-IL24-NKG2D-Exo suspension was added to each well and incubated for 24 h at 37 °C to allow for exosome uptake. After the 24 h co-culture, the medium was aspirated, and the cells were washed three times with DPBS. The cells were fixed in 4% PFA for 15 min, then washed three times with DPBS. The cells were permeabilized with 0.1% Triton X-100 in PBS for 10 min, followed by washing three times with DPBS. The cells were blocked with blocking buffer (5% BSA in DPBS) for 1 h at room temperature, then incubated with the anti-IL24 antibody (Clone Clound IL24 PAC064Hu01, Wuhan, China) overnight at 4 °C. After washing three times with DPBS, the cells were incubated with a fluorochrome-conjugated secondary antibody (AiFang biological AFSA005, Changsha, China) for 1 h in the dark and washed with DPBS. DAPI solution was added to each well for 5 min to stain the nuclei. The cells were visualized using a confocal microscope. The microscope filters were set to detect Dil, IL24 (green fluorescence), and DAPI.

### 4.12. Flow Cytometry for Tumor Cell Apoptosis

A549 and HELA cells were seeded and incubated for 24 h. An exosomes suspension was added to each well, and the cells were incubated at 37 °C for 24 and 48 h. After 24 h and 48 h, the mediums were collected, and the cells were washed by DPBS, which also were collected. Each well was added TrypLE™ Express Enzyme (Gibco, Grand Island, NY, USA) to detach the cells. The apoptosis of cells was detected by an apoptosis detection kit (Vazyme A213-02, Nanjing, China) according to the manufacturer’s protocol, and untreated cells were used positive control solutions (Multi Sciences AP-100-PCS, Hangzhou, China) to be a positive control.

### 4.13. CCK-8 Assay for Tumor Cell Growth Inhibition

A549, HELA, MCF-7, and 293T cells were seeded into 96-well plates and incubated for 24 h. The mediums were aspirated and washed with DPBS. Exosomes suspension was added to each well, and the cells were incubated at 37 °C for 12 h, 24 h, and 48 h to assess time-dependent induction of cell growth inhibition. After the 12 h, 24 h, and 48 h incubation periods, each well was added to CCK-8 reagent (Vazyme A311-02, Nanjing, China) and incubated for 1 h at 37 °C to measure the absorbance at 450 nm. The percentage of cell growth inhibition for each sample was calculated using the following formula:

$$\mathrm{Cell}\;\mathrm{viability}\;\left(\%\right)=\left(\frac{{\mathrm{OD}}_{\mathrm{sample}}-{\mathrm{OD}}_{\mathrm{blank}}}{{\mathrm{OD}}_{\mathrm{control}}-{\mathrm{OD}}_{\mathrm{control}}}\right)\times 100$$

$$\mathrm{Growth}\;\mathrm{inhibition}\;\mathrm{rate}\;\left(\%\right)=100-\mathrm{Cell}\;\mathrm{viability}\;(\%)$$

### 4.14. Scratch Assay to Assess the Impact of NK-EXO on Tumor Cell Migration

A549 cells were seeded and incubated for 24–48 h until they formed a confluent monolayer. The culture medium was carefully aspirated from each well, and using a sterile 200 µL pipette tip, a straight scratch (wound) was created down the center of the well by gently scraping through the cell monolayer. Any debris and floating cells were removed by washing the well twice with DPBS. For control wells, serum-free DMEM was added without exosomes. The cells were incubated at 37 °C for up to 48 h, capturing images at 0 h, 12 h, 36 h, and 48 h. Immediately after creating the scratch (0 h), images of the wound area were taken using an inverted phase-contrast microscope. At least three representative images were captured of the wound for each well. At 12 h, 36 h, and 48 h post-scratching, images of the same wound area were taken using the same microscope settings. Image J was used to measure the width of the wound at multiple points across the scratch.

Cell migration rate (%)=((Wound width at 0 h − Wound width at time))/(Wound width at 0 h)×100

### 4.15. Statistical Analysis

Data analyses were performed using Prism 9 (GraphPad) software. Data are presented as the mean ± standard deviation (SD). For all experiments, the significance of multiple-group comparisons was determined using a one-way ANOVA followed by Tukey’s test, with a *p*-value of less than 0.05 being considered significant.

## 5. Conclusions

In conclusion, this study elucidates the significant role of NK92MI-derived exosomes overexpressing NKG2D and IL24 in enhancing anti-tumor efficacy against various cancer cell lines, including A549, HELA, and MCF-7. The engineered exosomes demonstrated a superior capacity for inducing apoptosis, inhibiting cell proliferation, and preventing migration of tumor cells compared with control groups. The synergistic effects of NKG2D and IL24 underscore the potential of these exosomes as a promising therapeutic strategy for cancer immunotherapy. These findings warrant further exploration into the development of exosome-based therapies that can be tailored to enhance anti-tumor responses in diverse malignancies. Overall, the study contributes valuable insights into the potential applications of NK92MI cell-derived exosomes in oncotherapy, paving the way for future investigations into their therapeutic efficacy and mechanisms of action.

## Data Availability

The data are contained within the article and Supplementary Materials; further inquiries can be directed at the corresponding author.

## Supplementary Materials

The following supporting information can be downloaded at: https://www.mdpi.com/article/10.3390/ijms26052098/s1.
