# Supplementary material for: Overexpression of NKG2D and IL24 in NK Cell-Derived Exosomes for Cancer Therapy
Source: Int J Mol Sci. 2025 Feb 27;26(5):2098. doi: 10.3390/ijms26052098 (PMC11901126; doi:10.3390/ijms26052098)
Supplement: Supplementary file 1 [file ijms-26-02098-s001.zip › Figure S1.pdf]

A

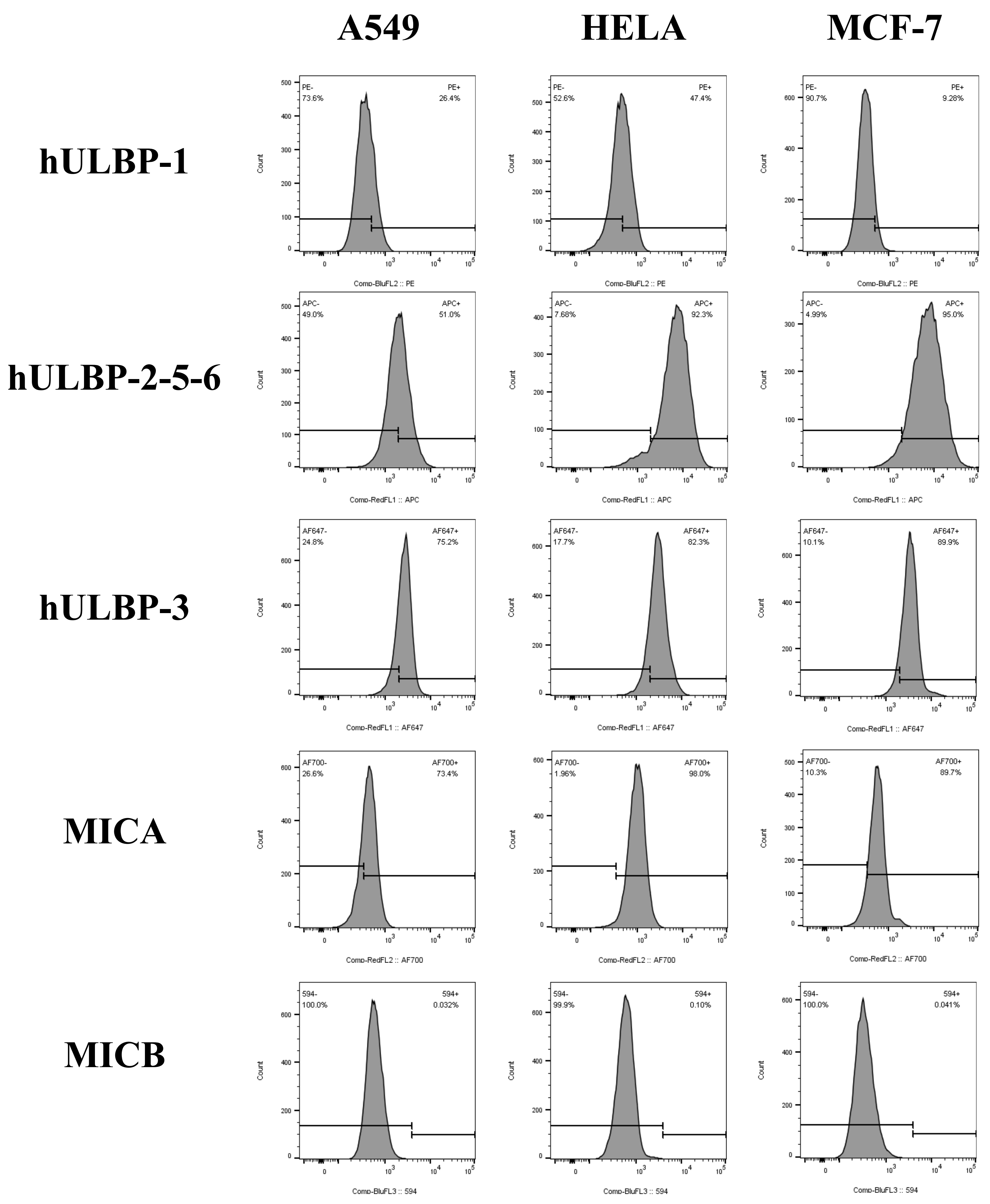

B

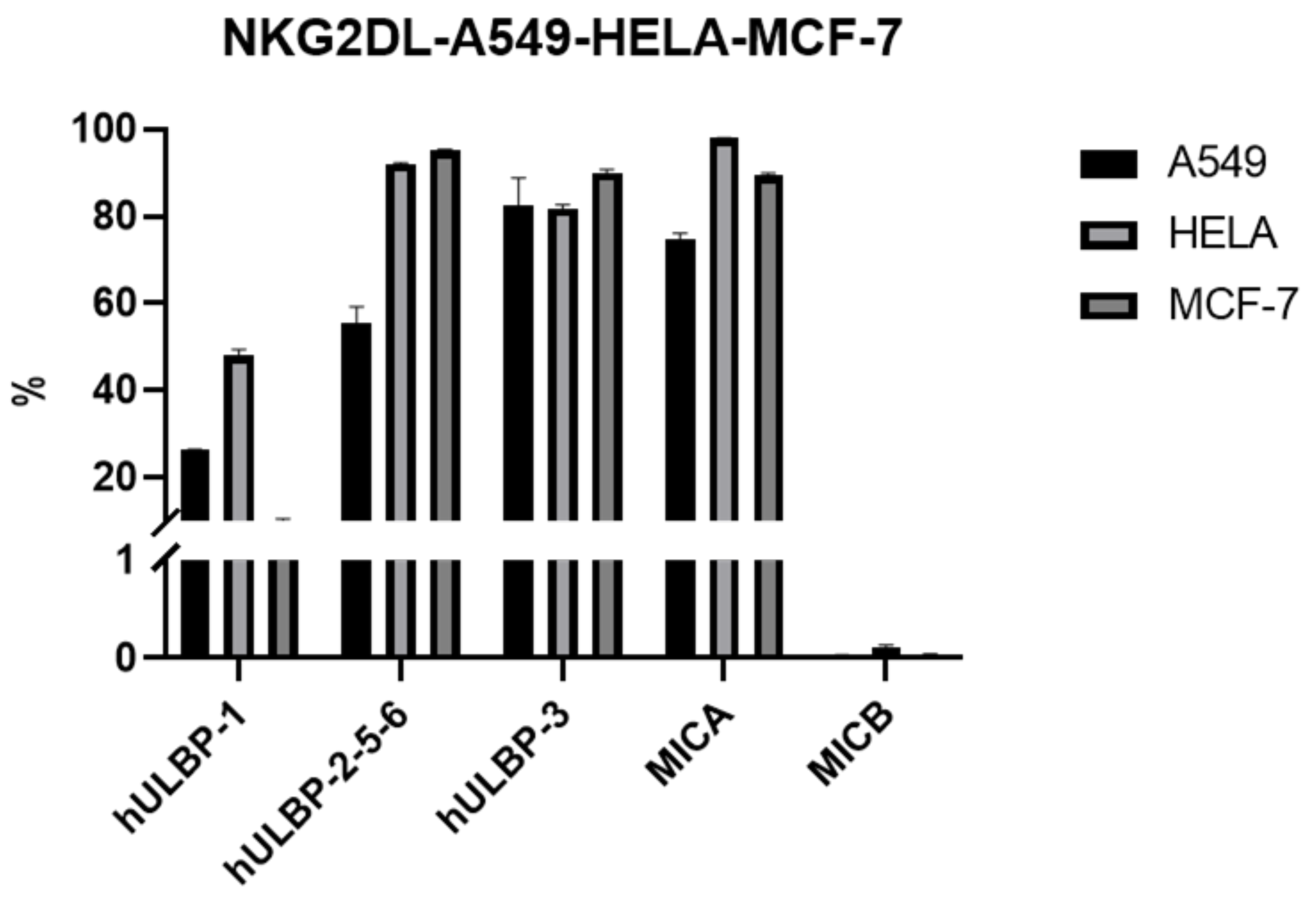

Figure S1. Flow Cytometry Detection of NKG2D Ligand Expression in Tumor Cells.
(A) Flow Cytometric Analysis of NKG2D Ligand in A549, HELA, MCF-7, (B) Positive proportion of NKG2D Ligand in A549, HELA, MCF-7.
