# Supplementary material for: Overexpression of NKG2D and IL24 in NK Cell-Derived Exosomes for Cancer Therapy
Source: Int J Mol Sci. 2025 Feb 27;26(5):2098. doi: 10.3390/ijms26052098 (PMC11901126; doi:10.3390/ijms26052098)
Supplement: Supplementary file 1 [file ijms-26-02098-s001.zip › Tables S1, S2 Figure S2.pdf]

Supplementary information.

Table S1. Nano-Flow Cytometry for the Purity and Diameter of Exosomes

	Particles(num)	TRITON-37℃-30min-particles(num)	purity% [1]	diameter(nm)
Blank-Exo	11093	2114	81.47	78.14±17.37
NKG2D-Exo	5754	985	83.91	80.16±18.01
IL24-NKG2D-Exo	3971	834	80.48	76.00±18.21
DPBS	61	59		

[1] Purity%=((Exo (Particles)-DPBS (Particles))-(Exo (TRITON-37 ℃-30 min-particles)-DPBS (Particles)-DPBS (TRITON-37 ℃-30 min-particles)))/ Exo (Particles)×100

Table S2. The Sequences of Primers

Primer	Forward	Reverse
IL24	5′-CAACTGCAACCCAGTCAAGAAA-3′	5′-TGCTCTCCGGAATAGCAGAAA-3′
NKG2D	5′-AGAGCCAGGCTTCTTGTATGT-3′	5′-GAATGGAGCCATCTTCCCACT-3′
GAPDH	5′-GGGGAGCCAAAAGGGTCATCATCT-3′	5′-GACGCCTGCTTCACCACCTTCTTG-3′

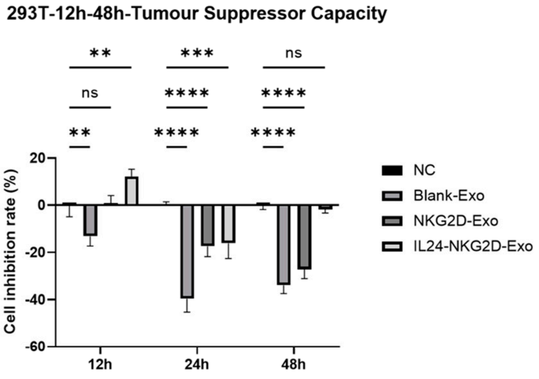

Figure S2. CCK-8 Assay for Growth Inhibition of Non-tumour Cells 293T Incubated with Exosomes at 12 hours, 24 hours and 48 hours. (significance was determined using a one-way ANOVA followed by Tukey's test; ns: not significant, ** $p < 0.01$, *** $p < 0.001$, **** $p < 0.0001$)
